# Duck Hunters’ Perceptions of Risk for Avian Influenza, Georgia, USA

**DOI:** 10.3201/eid1608.100032

**Published:** 2010-08

**Authors:** Hope Dishman, David Stallknecht, Dana Cole

**Affiliations:** Georgia Department of Community Health, Atlanta, Georgia, USA (H. Dishman, D. Cole); University of Georgia, Athens, Georgia, USA (D. Stallknecht, D. Cole); 1 Current affiliation: Centers for Disease Control and Prevention, Atlanta, Georgia, USA.

**Keywords:** Influenza A virus, influenza, duck hunter knowledge, attitudes, and practice, viruses, Georgia, dispatch

## Abstract

To determine duck hunters’ risk for highly pathogenic avian influenza, we surveyed duck hunters in Georgia, USA, during 2007–2008, about their knowledge, attitudes, and practices. We found they engage in several practices that could expose them to the virus. Exposures and awareness were highest for those who had hunted >10 years.

Introduction of highly pathogenic avian influenza virus (H5N1) (HPAI) could have several devastating effects in the United States. Illness and death caused by HPAI have been reported for humans, waterfowl, and other animals (*1*). In 2009, the estimated population of ducks susceptible to HPAI in the traditional survey area of North America was 42 million (*2*). Domestic poultry are also susceptible to HPAI (*1*). The retail equivalent of the broiler industry (which accounts for most commercial chicken production) in the United States was $44 billion in 2008; in 2007, Georgia led the country by producing 16% of all broilers (*3*).

Waterfowl and shorebirds are natural reservoirs of influenza A viruses (*4*). Antibodies to avian influenza virus (H11N9) have been detected in 2 of 68 Iowa Department of Natural Resources employees and in 1 of 39 Iowa duck hunters (*5*). These 3 men had 27, 30, and 31 years of experience, respectively, possibly indicating time- or behavior-dependent associations with exposure. We therefore sought to gain a better understanding of the knowledge, attitudes, and hunting practices of duck hunters and to better characterize their potential for exposure to influenza virus while hunting North American waterfowl. We hypothesized that the recent focus on the potential for introduction of HPAI into a North American migratory bird flyway (*6*) may increase hunter awareness of this virus.

## The Study

From November 17, 2007, through March 27, 2008, a convenience sample of 192 participants across the state of Georgia, USA, were surveyed in person (Technical Appendix). Participants included 61 active duck hunters in a wildlife management area and 131 members of Ducks Unlimited. Duck hunters at the wildlife management area were asked to complete a survey as they finished a morning of hunting. Ducks Unlimited members were approached at several of their banquets around Georgia and were asked to complete a survey if they were active duck hunters.

Analyses of survey data were conducted by using SAS software version 9.1 (SAS Institute Inc., Cary, NC, USA). Results across study groups were compared by using *t* tests, Mann-Whitney tests, and prevalence odds ratios (PORs). Statistical results were determined to be significant at p<0.05. This study was approved by the Georgia Department of Community Health Institutional Review Board.

To determine differences between those who were and were not members of Ducks Unlimited, we evaluated results from wildlife management area participants separately. A total of 37 (61%) wildlife management area participants reported that they were currently, or had been within the past 5 years, a member of Ducks Unlimited. Compared with nonmembers, members hunted more often per season—an average of 9.1× (95% confidence interval [CI] 2.0–16.2, p = 0.012) more than nonmembers. In addition, Ducks Unlimited members were 2.8× (95% CI 1.1–7.4, p = 0.033) more likely to have >10 years of hunting experience. Because Ducks Unlimited members did not differ significantly from nonmembers with regard to any other knowledge, attitude, or practice variable, we combined results of the wildlife management area survey with those of the Ducks Unlimited member survey.

In terms of hunting patterns and practices (Tables 1, 2), most (68%) hunters reported having hunted outside Georgia in the past 5 years. The 5 most common states visited for duck hunting outside of Georgia—from most to least common—were Arkansas, Alabama, Mississippi, Louisiana, and North Dakota. Experienced hunters (those with >10 years of hunting experience) reported hunting an average of 3.2 days more per season than those who had been hunting <10 years (p = 0.03). Experienced hunters were also significantly more likely to hunt outside of Georgia (POR 1.92, 95% CI 1.02–3.60, p = 0.042).

**Table 1:** Experience and hunting practices among surveyed duck hunters, Georgia, USA, November 17, 2007–March 27, 2008

Hunting practice	All hunters, no. (%)			<10 y, no. (%)			>10 y, no. (%)		Prevalence OR (95% CI)*	p value
	Yes	No		Yes	No		Yes	No		
Hunted outside Georgia in past 5 y, n = 190	130 (68)	60 (32)		64 (62)	39 (38)		66 (76)	21 (24)	1.92 (1.02–3.60)	0.042
Have direct contact with water, n = 190	173 (91)	17 (9)		91 (88)	12 (12)		82 (94)	5 (6)	2.16 (0.73–6.40)	0.156
Submerge head, n = 190	68 (36)	122 (64)		26 (25)	77 (75)		42 (48)	45 (52)	2.76 (1.50–5.10)	0.001
Always or occasionally use dog while hunting, n = 192	146 (76)	46 (24)		73 (70)	32 (30)		73 (84)	13 (16)	2.29 (1.13–4.63)	0.020
Process harvested duck (dress out), n = 190	165 (87)	25 (13)		88 (85)	15 (15)		77 (89)	10 (11)	1.31 (0.56–3.09)	0.533
Pluck and gut duck	73 (51)	69 (49)		34 (45)	41 (55)		39 (58)	28 (42)	1.68 (0.86–3.27)	0.125
Cut off breast muscle only	138 (88)	18 (12)		74 (87)	11 (13)		64 (90)	7 (10)	1.36 (0.50–3.71)	0.549
Wear gloves while dressing out, n = 163	26 (16)	137 (84)		15 (17)	71 (83)		11 (14)	66 (86)	0.79 (0.34–1.84)	0.583
Take ducks to taxidermist, n = 190	113 (59)	77 (41)		50 (49)	53 (51)		63 (72)	24 (28)	2.78 (1.51–5.11)	0.001
Share harvested ducks, n = 188	118 (63)	70 (37)		63 (62)	38 (38)		55 (63)	32 (37)	1.04 (0.57–1.88)	0.905
Dress ducks before sharing, n = 115	62 (54)	53 (46)		36 (60)	24 (40)		26 (47)	29 (53)	0.60 (0.29–1.25)	0.171
Consume meat from harvested ducks, n = 187	176 (94)	11 (6)		93 (93)	7 (7)		83 (95)	4 (5)	1.56 (0.44–5.53)	0.486
Know that others consume meat from harvest, n = 188	144 (77)	44 (23)		74 (73)	27 (27)		70 (80)	17 (20)	1.50 (0.75–2.99)	0.246

*Referent group for calculation is duck hunters who reported <10 y of experience duck hunting. OR, odds ratio; CI, confidence interval.

**Table 2:** Duck hunter experience and exposure to avian influenza (H5N1), Georgia, USA, November 17, 2007–March 27, 2008

Hunters	Years of hunting, no. (%) hunters, n = 189					Exposure, mean (range)		
	1	2–5	6–10	>10		Hunts/y, n = 191*	Harvests/y, n = 188†	Ducks sent to taxidermist in past 5 y, n = 108‡
All	9 (5)	42 (22)	51 (27)	87 (46)		14.8 (1–60)	26.2 (1–240)	5.6 (1–60)
Hunted <10 y	9 (9)	42 (41)	51 (50)	0		13.4 (1–60)	24.3 (1–240)	5.6 (1–60)
Hunted >10 y	0	0	0	87 (100)		16.5 (2–60)	28.3 (2–200)	5.6 (1–30)

*Differences in mean values, calculated by Mann-Whitney U test, were –3.2 (p = 0.03). †Differences in mean values, calculated by Mann-Whitney U test, were –4.0 (p = 0.08). ‡Differences in mean values, calculated by Mann-Whitney U test, were 0.01 (p = 0.03).

Almost all (91%) hunters reported having had direct contact with water while hunting. Experienced hunters were significantly more likely to report having submerged their head in water during a hunt (POR 2.76, 95% CI 1.50–5.10, p = 0.001). Most (87%) hunters processed their harvested ducks themselves, and 84% did not wear gloves while doing so. However, most (88%) somewhat limited their postharvest exposures by leaving most of the bird intact and simply cutting the breast meat from the carcass; only 51% reported completely dressing out the duck by plucking and gutting the carcass. Awareness of HPAI infection, or bird flu, was common among duck hunters (86%), but knowledge of the signs and symptoms in infected humans was not (23%). Only 6 (3%) respondents said that they would stop hunting if HPAI were found in US duck populations, and 36 (19%) would stop duck hunting if the virus were found in the state of Georgia.

Experienced hunters were nearly 3× more likely than less experienced hunters to have heard of HPAI (POR = 2.72, 95% CI 1.09–6.78, p = 0.027). However, experienced hunters who reported this virus as a personal concern said that they were not more likely to change their hunting practices if it were found in the United States or Georgia. Unlike the experienced hunters, less experienced hunters who reported concern about HPAI were 7.5× more likely to stop hunting if the virus were found in ducks in Georgia (95% CI 2.08–27.02, p = 0.001); those who were concerned about their own risk for illness through contact with sick birds were 4.8× more likely to stop hunting if HPAI were found in ducks in Georgia (95% CI 1.70–13.59, p = 0.002).

## Conclusions

If HPAI were to become established in duck populations in North America, risk for human exposure to the virus through hunting could be substantial. In Georgia, each of the ≈12,000 active duck hunters (*7*) and ≈19,000 members of Ducks Unlimited potentially has contact with influenza-infected ducks and their water environments while hunting. By processing an influenza-infected duck, a hunter may be exposed to virus-laden nasal and/or fecal excretions in addition to blood, tissues, and other body fluids (*8*). Most hunters process harvested ducks themselves and do not use gloves. In the Republic of Azerbaijan, defeathering waterfowl infected with HPAI was associated with 8 confirmed cases of human illness and 5 deaths (*9*), but worldwide, no reports of HPAI infections among waterfowl hunters have been documented.

Influenza A viruses can persist in water for extended periods (*10*) and on clothing for several hours (*11*). Most hunters have direct contact with water during a hunt, but experienced hunters are more likely to have their head submerge. Although hunters could be exposed to virus during contact with contaminated water and by aerosols generated when water- or carcass-contaminated clothing are removed, no data are available to realistically evaluate this possibility.

To minimize duck hunter exposure to HPAI, we recommend more use of personal protection, such as gloves, while processing harvested ducks. If this virus were found in North America, increased efforts to educate duck hunters on the potential severity of illness resulting from HPAI and the potential for exposure during hunting might decrease risky hunting practices.

If HPAI were found in United States or Georgia duck populations, most duck hunters indicated that they would not stop hunting. These findings suggest that hunting practices and attitudes among the subpopulation of experienced hunters may contribute to an increased risk for avian influenza infection.

## Supplementary Material

Technical Appendix Attachment A: Hunter Survey.
