# Supplementary material for: Duck Hunters’ Perceptions of Risk for Avian Influenza, Georgia, USA
Source: Emerg Infect Dis. 2010 Aug;16(8):1279–81. doi: 10.3201/eid1608.100032 (PMC3298320; doi:10.3201/eid1608.100032)
Supplement: Technical Appendix — Attachment A: Hunter Survey. [file 10-0032-Techapp.pdf]

Article DOI: 10.3201/eid1608.100032

Attachment A: Hunter Survey

The Georgia Division of Public Health is conducting a study of the human exposures to waterfowl species in Georgia at risk of avian influenza infection. Your participation is very important to the success of this study. The information collected will be summarized and used to develop specific surveillance and outreach activities to improve avian influenza prevention and response activities in Georgia. The information collected in this study will in no way impact your license or relationship with the Georgia Department of Natural Resources. If you have any questions or concerns about your participation in this survey, please contact Dr. Dana Cole, Georgia Public Health Veterinarian, at 404-657-2593.

Please read the questions thoroughly and answer them to the best of your ability.

A. Hunting Experience

1. Where do you duck hunt in Georgia? (Check all that apply)

☐ Major Reservoir ____
(Name of Reservoir, e.g. Seminole, Juliette, Clarks Hill, etc.)

☐ Coastal Marsh ____
(County or Wildlife Management Area, e.g. Altamaha WMA, etc.)

☐ Public Lands ____
(WMA or National Forest, e.g. Oconee WMA, etc.)

☐ Hunting Preserve ____
(Name of Preserve(s) or Lodge(s))

☐ Private Pond ____
(Counties duck hunted in last year)

2. In the past 5 years, have you gone duck hunting outside of Georgia? ☐ Yes ☐ No
If yes, where have you gone? (List States)

____
(States)

3. How many times do you duck hunt during an average season?

☐ 1 ☐ 2 ☐ 3 ☐ 4 ☐ 5 ☐ 6 ☐ 7 ☐ 8 ☐ 9 ☐ 10 or more

If more than 10, how many times?__________________

Thank you for your participation in this survey.

4. How many years have you been hunting ducks?

☐ This is first year ☐ 2-5yrs ☐ 6-10yrs

If more than 10, how many years? ____________

5. Do you have direct contact with the water while you duck hunt? For example, do you get your hands or face wet?

☐ Yes ☐ No

6. Has your head ever been submerged (gone under water) while duck hunting?

☐ Yes ☐ No **If yes**, about how many times per season? _______________

7. Have you ever accidentally or intentionally ingested (swallowed) any river/lake water while duck hunting either? ☐Yes

☐ No ☐ Not Sure

8. Do you use a dog while duck hunting? ☐Always ☐ Occasionally ☐ Never

B. Harvest

9. How many ducks do you harvest in an average year?

☐ 1 ☐ 2 ☐ 3 ☐ 4 ☐ 5 ☐ 6 ☐ 7 ☐ 8 or more

If more than 8, how many ducks?___________________________

10. Do you dress your own ducks? ☐Yes ☐ No

If yes,

a. Do you pluck and gut the duck? ☐Yes ☐ No

b. Or, do you just fillet the breasts off? ☐Yes ☐ No

c. Do you wear gloves while dressing the duck? ☐Yes ☐ No

11. If you do not dress your own ducks, do you take them to a processing facility to have them dressed? ☐Yes ☐ No

12. Have you ever taken your ducks to a taxidermist? ☐Yes ☐ No

If yes, how many ducks have you taken in the past 5 years?

☐ 1 ☐ 2 ☐ 3 ☐ 4 ☐ 5 ☐ 6 ☐ 7 ☐ 8 or more

Thank you for your participation in this survey.

If more than 8, how many ducks?______________________________

13. Do you give any of your harvested ducks to other people? ☐Yes ☐ No

    If yes, are they dressed before you give them away? ☐Yes ☐ No

14. Do you consume the meat from your harvested ducks? ☐Yes ☐ No

15. Do others in your family consume the meat from your harvested ducks?

    ☐Yes ☐ No **If yes**, how many others on average? _______________

D. Flu Awareness

16. Do you worry about catching a flu each winter? ☐Yes ☐ No

17. Do you usually get a seasonal flu shot every year? ☐Yes ☐ No ☐ Not Sure

18. Have you ever heard of avian influenza or "bird flu?" ☐Yes ☐ No
    If yes, do you know the signs and symptoms of avian influenza in people?

    ☐Yes ☐ No ☐ Not Sure

19. Is exposure to avian influenza ("bird flu") a personal concern for you right now?

    ☐Yes ☐ No

20. Are you concerned about being at risk of illness if you come in contact with sick ducks while hunting? ☐Yes ☐ No

21. If you learned that avian influenza ("bird flu") was found in ducks in the U.S. would you stop hunting ducks?

    ☐Yes ☐ No ☐ Only if infected ducks were found in Georgia

Thank you for your participation in this survey.
